# Supplementary material for: Antineoplastic Activity of Rhus trilobata Nutt. (Anacardiaceae) against Ovarian Cancer and Identification of Active Metabolites in This Pathology
Source: Plants (Basel). 2021 Sep 30;10(10):2074. doi: 10.3390/plants10102074 (PMC8540642; doi:10.3390/plants10102074)
Supplement: Supplementary file 1 [file plants-10-02074-s001.zip › plants-1372441-SI.pdf]

plants

MDPI

Supplementary Materials

1. Supplementary Figures

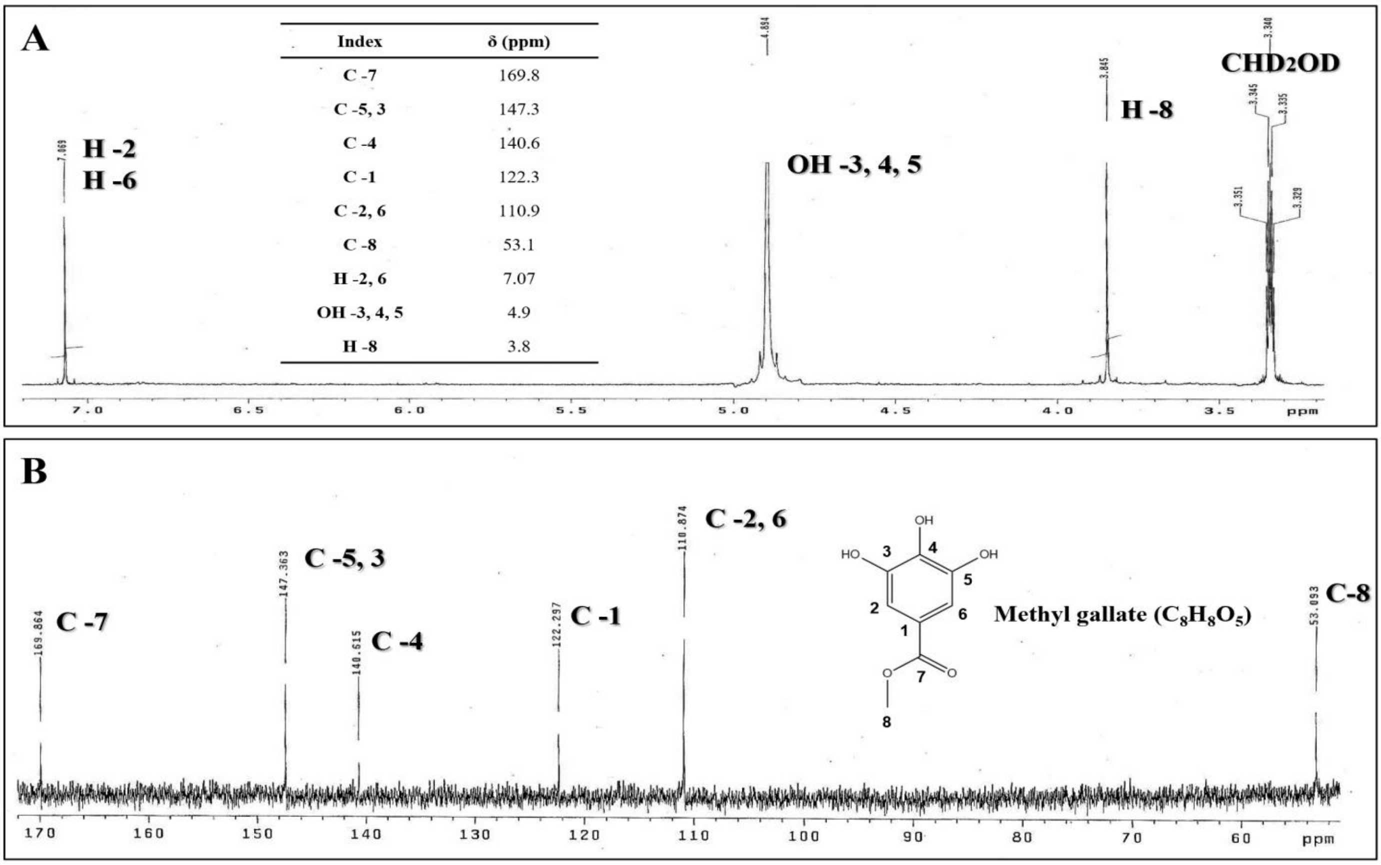

Figure S1. Spectrum of methyl 3,4,5-trihydroxybenzoate ($C_8H_8O_5$) by NMR at 300 MHz. The figure shows the chemical structure and δ (ppm) of the compound. ^{1}H-NMR (**A**) and ^{13}C-NMR (**B**). Results of three biological replicates (n = 3, in triplicates). The compound was putatively identified by the fragmentation pattern and metabolic databases (AraCyc, PlantCyc, and KEGG).

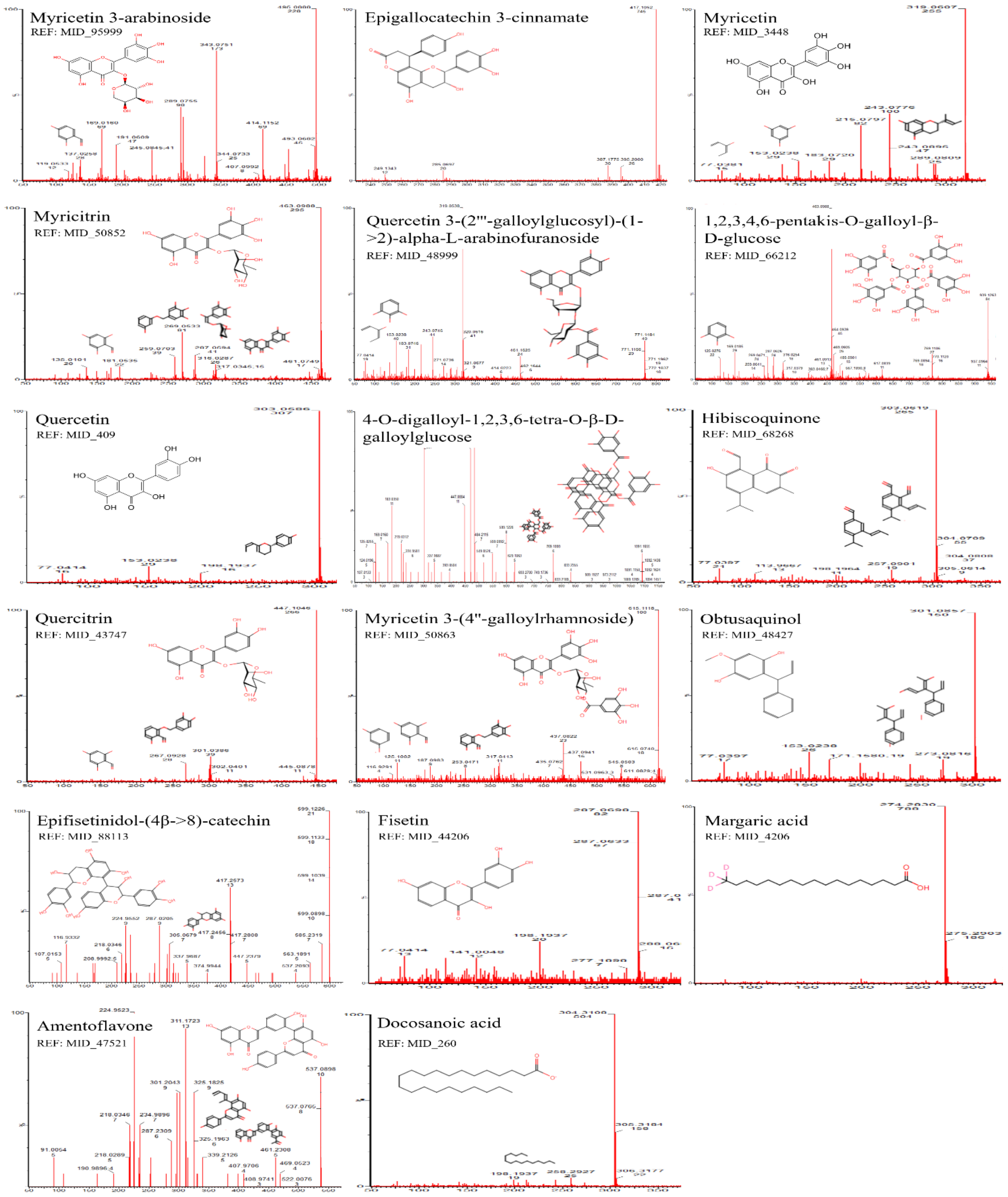

Figure S2. MS/MS analysis of compounds most abundant in AF02-RHTR. Figure show fragmentation pattern utilized for putatively identified the compounds most abundant in AF02, by comparison with reports in plant metabolomics databases with Progenesis® QI. The inset show fragmentation of standard compounds used for corroborating the identification.

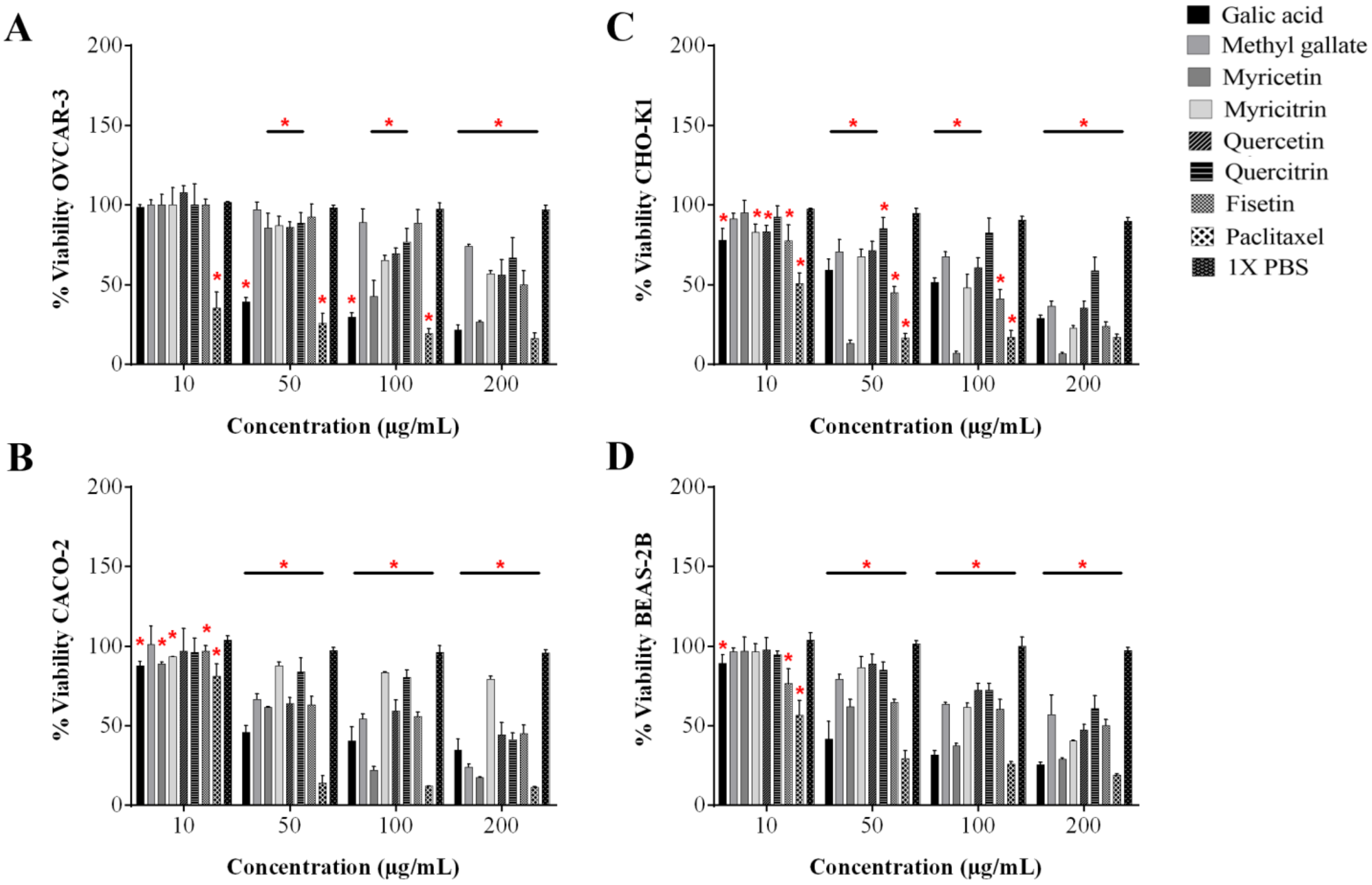

Figure S3. Biological activity of metabolites in RHTR into ovarian cancer and normal cell lines. The IC_{50} was determined by dose-response viability curves with MTT for 24 h in OVCAR-3 (**A**), CACO-2 (**B**), CHO-K1 (**C**) and BEAS-2B (**D**) cells. Results show the mean ± S.D. of three biological replicates (*n* = 3, in triplicates); *, $p \leq 0.05$ vs. the vehicle group without treatment (0.5 % DMSO) (ANOVA). The positive control was paclitaxel.

2. Supplementary Tables

Table S1. Morphometric analysis of liver and kidneys in *Nu*/*Nu* mice treated with RHTR.

Treatments	Liver	Kidneys
AE	1.48 ± 0.22 / 29.41 ± 4.06	0.38 ± 0.048 / 9.63 ± 0.69
AF02	1.61 ± 0.15 / 30.34 ± 3.33	0.40 ± 0.06 / 9.89 ± 0.58
Carboplatin	1.80 ± 0.10 * / 30.5 ± 2.2	0.40 ± 0.01 / 11.5 ± 0.7 *
1X PBS	1.53 ± 0.14 / 29.65 ± 2.22	0.38 ± 0.042 / 9.34 ± 0.67

Results show the mean ± S.D. of two biological replicates (n = 5).
Measurement of weight (g) and larger diameter (mm) for liver and kidney
*, $p \leq 0.05$ vs. control values treated with 1X PBS vehicle (ANOVA).
The positive control was carboplatin (50 mg/kg/i.p./3 alternating days per week in mice).
RHTR, *Rhus trilobata*; AE, aqueous extract; AF02, aqueous fraction-02 (active fraction).
